# Supplementary material for: Hybrid Models and Biological Model Reduction with PyDSTool
Source: PLoS Comput Biol. 2012 Aug 9;8(8):e1002628. doi: 10.1371/journal.pcbi.1002628 (PMC3415397; doi:10.1371/journal.pcbi.1002628)
Supplement: Text S4 — Complete source code for the PyDSTool package (version 0.88.120504). Includes API documentation and help files linking to web pages. This file is identical to the current public release on Sourceforge.net. (ZIP) [file pcbi.1002628.s004.zip › PyDSTool/html/PyDSTool.Toolbox.phaseplane.Point2D-class.html]

xml version="1.0" encoding="ascii"?


PyDSTool.Toolbox.phaseplane.Point2D


| Home | Trees | Indices | Help | PyDSTool |
| --- | --- | --- | --- | --- |

Package PyDSTool ::
Package Toolbox ::
Module phaseplane ::
Class Point2D

[hide private]

[frames] | no frames]

# Class Point2D

source code

```
  object --+    
           |    
Points.Point --+
               |
              Point2D
```

---

Convenience sub-class of PyDSTool.Point for 2D Euclidean points. If
initialized using a dictionary, xname and yname optional arguments must
specify which coordinate is associated with 'x' and 'y' axes.

Main advantage is lower overhead of initialization and the more
convenient notation point.x and point.y for the two coordinates, that may
nonetheless be given optional actual names (xname and yname). Note that
Python's "dot-based" attribute lookup is only valid for 'x' and
'y' fields.

Optional norm order and labels argument still permitted, as for
original Point. For 2D Pointsets, use original PyDSTool.Pointset
class.


|  |  |
| --- | --- |
| Instance Methods | [hide private] |

|  |  |
| --- | --- |
| \_\_init\_\_(self, x, y=None, xname=`'``x``'`, yname=`'``y``'`, norm=2, labels=None)  If y is None, expects 2D array or dictionary in x | source code |

|  |  |
| --- | --- |
| mapNames(self, themap)  Map coordinate names and label(s), using a symbol map of class symbolMapClass. | source code |

|  |  |
| --- | --- |
| \_\_contains\_\_(self, coord) | source code |

|  |  |
| --- | --- |
| todict(self, aslist=False)  Convert Point2D to a dictionary of array values (or of list with aslist=True). | source code |

|  |  |
| --- | --- |
| toarray(self) | source code |

|  |  |
| --- | --- |
| toPoint(self)  Coerce to regular PyDSTool.Point object | source code |

|  |  |
| --- | --- |
| get(self, coord, d=None) | source code |

|  |  |
| --- | --- |
| \_\_len\_\_(self) | source code |

|  |  |
| --- | --- |
| \_\_abs\_\_(self) | source code |

|  |  |
| --- | --- |
| update(self, d) | source code |

|  |  |
| --- | --- |
| items(self) | source code |

|  |  |
| --- | --- |
| iteritems(self) | source code |

|  |  |
| --- | --- |
| values(self) | source code |

|  |  |
| --- | --- |
| itervalues(self) | source code |

|  |  |
| --- | --- |
| keys(self) | source code |

|  |  |
| --- | --- |
| iterkeys(self) | source code |

|  |  |
| --- | --- |
| has\_key(self, k) | source code |

|  |  |
| --- | --- |
| \_\_getitem\_\_(self, ix) | source code |

|  |  |
| --- | --- |
| \_\_call\_\_(self, ix) | source code |

|  |  |
| --- | --- |
| \_\_setitem\_\_(self, ix, val)  Change coordinate array values. | source code |

|  |  |
| --- | --- |
| \_\_delitem\_\_(self, k) | source code |

|  |  |
| --- | --- |
| \_\_str\_\_(self)  str(x) | source code |

|  |  |
| --- | --- |
| \_\_repr\_\_(self)  str(x) | source code |

|  |  |
| --- | --- |
| info(self, verboselevel=1) | source code |

|  |  |
| --- | --- |
| \_\_copy\_\_(self) | source code |

|  |  |
| --- | --- |
| \_\_add\_\_(self, other) | source code |

|  |  |
| --- | --- |
| \_\_sub\_\_(self, other) | source code |

|  |  |
| --- | --- |
| \_\_radd\_\_(self, other) | source code |

|  |  |
| --- | --- |
| \_\_rsub\_\_(self, other) | source code |

|  |  |
| --- | --- |
| \_\_mul\_\_(self, c) | source code |

|  |  |
| --- | --- |
| \_\_div\_\_(self, c) | source code |

|  |  |
| --- | --- |
| \_\_rmul\_\_(self, c) | source code |

|  |  |
| --- | --- |
| \_\_rdiv\_\_(self, c) | source code |

|  |  |
| --- | --- |
| \_\_truediv\_\_(self, c) | source code |

|  |  |
| --- | --- |
| \_\_rtruediv\_\_(self, c) | source code |

|  |  |
| --- | --- |
| \_\_neg\_\_(self) | source code |

|  |  |
| --- | --- |
| \_\_pow\_\_(self, other) | source code |

|  |  |
| --- | --- |
| \_\_eq\_\_(self, other) | source code |

|  |  |
| --- | --- |
| \_\_lt\_\_(self, other) | source code |

|  |  |
| --- | --- |
| \_\_gt\_\_(self, other) | source code |

|  |  |
| --- | --- |
| \_\_le\_\_(self, other) | source code |

|  |  |
| --- | --- |
| \_\_ge\_\_(self, other) | source code |

**Inherited from `Points.Point`**:
`__getstate__`,
`__ne__`,
`__pos__`,
`__setstate__`,
`addlabel`,
`copy`,
`makeIxMaps`,
`removelabel`

**Inherited from `Points.Point`** (private):
`_force_coords_to_ixlist`,
`_infostr`,
`_map_names_to_ixs`

**Inherited from `object`**:
`__delattr__`,
`__getattribute__`,
`__hash__`,
`__new__`,
`__reduce__`,
`__reduce_ex__`,
`__setattr__`


|  |  |
| --- | --- |
| Properties | [hide private] |

**Inherited from `object`**:
`__class__`


|  |  |
| --- | --- |
| Method Details | [hide private] |

|  |  |
| --- | --- |
| \_\_init\_\_(self, x, y=None, xname=`'``x``'`, yname=`'``y``'`, norm=2, labels=None)  *(Constructor)* | source code |

If y is None, expects 2D array or dictionary in x

Overrides: object.\_\_init\_\_

|  |  |
| --- | --- |
| mapNames(self, themap) | source code |

Map coordinate names and label(s), using a symbol map of class
symbolMapClass.

Overrides: Points.Point.mapNames: *(inherited documentation)*

|  |  |
| --- | --- |
| \_\_contains\_\_(self, coord)  *(In operator)* | source code |

Overrides: Points.Point.\_\_contains\_\_

|  |  |
| --- | --- |
| todict(self, aslist=False) | source code |

Convert Point2D to a dictionary of array values (or of list with
aslist=True).

Overrides: Points.Point.todict

|  |  |
| --- | --- |
| toarray(self) | source code |

Overrides: Points.Point.toarray

|  |  |
| --- | --- |
| get(self, coord, d=None) | source code |

Overrides: Points.Point.get

|  |  |
| --- | --- |
| \_\_len\_\_(self)  *(Length operator)* | source code |

Overrides: Points.Point.\_\_len\_\_

|  |  |
| --- | --- |
| \_\_abs\_\_(self) | source code |

Overrides: Points.Point.\_\_abs\_\_

|  |  |
| --- | --- |
| update(self, d) | source code |

Overrides: Points.Point.update

|  |  |
| --- | --- |
| items(self) | source code |

Overrides: Points.Point.items

|  |  |
| --- | --- |
| iteritems(self) | source code |

Overrides: Points.Point.iteritems

|  |  |
| --- | --- |
| values(self) | source code |

Overrides: Points.Point.values

|  |  |
| --- | --- |
| itervalues(self) | source code |

Overrides: Points.Point.itervalues

|  |  |
| --- | --- |
| keys(self) | source code |

Overrides: Points.Point.keys

|  |  |
| --- | --- |
| iterkeys(self) | source code |

Overrides: Points.Point.iterkeys

|  |  |
| --- | --- |
| has\_key(self, k) | source code |

Overrides: Points.Point.has\_key

|  |  |
| --- | --- |
| \_\_getitem\_\_(self, ix)  *(Indexing operator)* | source code |

Overrides: Points.Point.\_\_call\_\_

|  |  |
| --- | --- |
| \_\_call\_\_(self, ix)  *(Call operator)* | source code |

Overrides: Points.Point.\_\_call\_\_

|  |  |
| --- | --- |
| \_\_setitem\_\_(self, ix, val)  *(Index assignment operator)* | source code |

Change coordinate array values.

Overrides: Points.Point.\_\_setitem\_\_: *(inherited documentation)*

|  |  |
| --- | --- |
| \_\_delitem\_\_(self, k)  *(Index deletion operator)* | source code |

Overrides: Points.Point.\_\_delitem\_\_

|  |  |
| --- | --- |
| \_\_str\_\_(self)  *(Informal representation operator)* | source code |

str(x)

Overrides: object.\_\_str\_\_: *(inherited documentation)*

|  |  |
| --- | --- |
| \_\_repr\_\_(self)  *(Representation operator)* | source code |

str(x)

Overrides: object.\_\_repr\_\_: *(inherited documentation)*

|  |  |
| --- | --- |
| info(self, verboselevel=1) | source code |

Overrides: Points.Point.info

|  |  |
| --- | --- |
| \_\_copy\_\_(self) | source code |

Overrides: Points.Point.\_\_copy\_\_

|  |  |
| --- | --- |
| \_\_add\_\_(self, other)  *(Addition operator)* | source code |

Overrides: Points.Point.\_\_add\_\_

|  |  |
| --- | --- |
| \_\_sub\_\_(self, other)  *(Subtraction operator)* | source code |

Overrides: Points.Point.\_\_sub\_\_

|  |  |
| --- | --- |
| \_\_radd\_\_(self, other)  *(Right-side addition operator)* | source code |

Overrides: Points.Point.\_\_add\_\_

|  |  |
| --- | --- |
| \_\_rsub\_\_(self, other) | source code |

Overrides: Points.Point.\_\_rsub\_\_

|  |  |
| --- | --- |
| \_\_mul\_\_(self, c) | source code |

Overrides: Points.Point.\_\_mul\_\_

|  |  |
| --- | --- |
| \_\_div\_\_(self, c) | source code |

Overrides: Points.Point.\_\_div\_\_

|  |  |
| --- | --- |
| \_\_rmul\_\_(self, c) | source code |

Overrides: Points.Point.\_\_mul\_\_

|  |  |
| --- | --- |
| \_\_rdiv\_\_(self, c) | source code |

Overrides: Points.Point.\_\_rdiv\_\_

|  |  |
| --- | --- |
| \_\_truediv\_\_(self, c) | source code |

Overrides: Points.Point.\_\_div\_\_

|  |  |
| --- | --- |
| \_\_rtruediv\_\_(self, c) | source code |

Overrides: Points.Point.\_\_rdiv\_\_

|  |  |
| --- | --- |
| \_\_neg\_\_(self) | source code |

Overrides: Points.Point.\_\_neg\_\_

|  |  |
| --- | --- |
| \_\_pow\_\_(self, other) | source code |

Overrides: Points.Point.\_\_pow\_\_

|  |  |
| --- | --- |
| \_\_eq\_\_(self, other)  *(Equality operator)* | source code |

Overrides: Points.Point.\_\_eq\_\_

|  |  |
| --- | --- |
| \_\_lt\_\_(self, other)  *(Less-than operator)* | source code |

Overrides: Points.Point.\_\_lt\_\_

|  |  |
| --- | --- |
| \_\_gt\_\_(self, other)  *(Greater-than operator)* | source code |

Overrides: Points.Point.\_\_gt\_\_

|  |  |
| --- | --- |
| \_\_le\_\_(self, other)  *(Less-than-or-equals operator)* | source code |

Overrides: Points.Point.\_\_le\_\_

|  |  |
| --- | --- |
| \_\_ge\_\_(self, other)  *(Greater-than-or-equals operator)* | source code |

Overrides: Points.Point.\_\_ge\_\_

  


| Home | Trees | Indices | Help | PyDSTool |
| --- | --- | --- | --- | --- |

|  |  |
| --- | --- |
| Generated by Epydoc 3.0.1 on Fri May 4 15:24:09 2012 | http://epydoc.sourceforge.net |
